# Geometry Control of Source/Drain Electrodes in Organic Field-Effect Transistors by Electrohydrodynamic Inkjet Printing

**DOI:** 10.3390/ma13214974

**Published:** 2020-11-05

**Authors:** Piotr Sleczkowski, Michal Borkowski, Hanna Zajaczkowska, Jacek Ulanski, Wojciech Pisula, Tomasz Marszalek

**Affiliations:** 1 Department of Molecular Physics, Faculty of Chemistry, Lodz University of Technology, Zeromskiego 116, 90-924 Lodz, Poland; michal.borkowski@dokt.p.lodz.pl (M.B.); hanna.zajaczkowska@dokt.p.lodz.pl (H.Z.); jacek.ulanski@p.lodz.pl (J.U.); pisula@mpip-mainz.mpg.de (W.P.); 2 Max Planck Institute for Polymer Research, Ackermannweg 10, 55128 Mainz, Germany

**Keywords:** printed electronics, electrohydrodynamic inkjet printing, organic field-effect transistors

## Abstract

In this work we study the influence of dielectric surface and process parameters on the geometry and electrical properties of silver electrodes obtained by electrohydrodynamic inkjet printing. The cross-section and thickness of printed silver tracks are optimized to achieve a high conductivity. Silver overprints with cross-section larger than 4 μm^2^ and thickness larger than 90 nm exhibit the lowest resistivity. To fabricate electrodes in the desired geometry, a sufficient volume of ink is distributed on the surface by applying appropriate voltage amplitude. Single and multilayer overprints are incorporated as bottom contacts in bottom gate organic field-effect transistors (OFETs) with a semiconducting polymer as active layer. The multilayer electrodes result in significantly higher electrical parameters than single layer contacts, confirming the importance of a careful design of the printed tracks for reliable device performance. The results provide important design guidelines for precise fabrication of electrodes in electronic devices by electrohydrodynamic inkjet printing.

## 1. Introduction

Micro- and nanofabrication of functional structures on surfaces is of the utmost importance for the development of numerous existing and emerging technologies in electronics [1,2], optics [3,4], medicine [5] and pharmaceuticals [6] among others [7]. The electronic and optoelectronic industries typically exploit lithographic methods to continuously increase the density of integrated circuits by size reduction of the components [8]. The pitch of (opto)electronic elements is precisely controlled down to the 100 nm scale by optical lithography [9]. However, the emergence of new research areas, especially at the interface with organic and biological materials, requires the development of fabrication methods employing conditions that are less demanding in terms of chemical and thermal processing. The subtractive character of conventional lithographic methods can cause, for example, degradation of materials under extreme conditions of temperature or pressure. To circumvent these constraints, alternative approaches based on additive fabrication are continuously being developed [10,11]. Inkjet printing is one of the most widely used additive fabrication methods which enables deposition of material of interest on various types of surface, including flexible and stretchable substrates [12]. This is particularly important from the perspective of development of wearable devices that can integrate seamlessly with the human body as e.g., sensors for health monitoring [13]. Inkjet printing is a non-contact method that relies on the generation of droplets from the aperture of the printing nozzle [14]. Most often it is realized with the use of pressure pulses generated by thermal or piezoelectric means [15]. The movement control of the printing nozzle (or the sample), together with the possibility of delivering the discrete amount of ink, allows pre-defined layouts of materials to be created. The achievable resolution of the inkjet printed patterns is determined by the volume of droplet ejected from the nozzle [14,15]. In the case of thermal/piezoelectric printheads the picoliter size droplets are deposited on the surface, resulting in printed features in the range of tens of micrometers [16]. Since capillary forces become more pronounced as the characteristic length shrinks, pushing out the droplet becomes more difficult as the nozzle orifice becomes smaller [17]. An alternative to pressure-driven droplet ejection is the use of the electric field which pulls the liquid out of the nozzle. Since the generation of an electrohydrodynamic jet is less dependent on the nozzle diameter, especially in the cone-jet mode where a Taylor cone is formed, the electrohydrodynamically induced flows offer an efficient way of improving the printing resolution with respect to conventional inkjet printing [18]. Over the past decade, electrohydrodynamic (e-jet) printing has attracted growing interest, especially in the areas of printed electronics [19], sensors [20], supercapacitors [21], photonic devices [22] and 3D printing [23]. Using e-jet printing for the fabrication of electronic components offers many advantages, particularly in the elaboration of source and drain electrodes for field-effect transistors (FETs), for which a precise control of shape is indispensable for ensuring reliable device performance. Regardless the droplet generation method, the first step to realize electrodes relies on the control of the deposited droplet morphology that promotes their coalescence and results in the formation of continuous lines [24]. Various strategies for tailoring the deposition morphologies include the suppressing (or utilizing) of the coffee-ring effect [25] and using patterned substrates [26]. Surface wettability influences significantly the feature size and cross-sectional profile [27] and determines the uniformity of the printed lines [28]. Besides the basic requirements of low resistance, good contact with the semiconductor and uniform morphology of printed lines are also particularly crucial for bottom contact organic FETs (OFETs). The morphology of the contact is important due to channel length variations resulting from lateral imperfections of the printed lines, while the ordering of the semiconductor molecules strongly depends on the surface topography of the electrode. Some of the principal printed line behaviours deviating from the ideal (uniform mode) are scalloped, bulging and stacked coins, whereby the morphology is altered by the drop spacing and substrate temperature [29]. The combination of drop spacing and substrate temperature reduces the coffee-ring effect and results in this way in an improved performance of inkjet-printed Ag source/drain electrodes in OFETs [30]. In the case of water-based inks, the Ag electrode profile can be tuned by controlling the ambient humidity [31]. Besides temperature and humidity, the modification of the surface energy of the substrate by self-assembled monolayers [32], ultraviolet (UV)-ozone [33] or plasma treatment [34] is established as an alternative way for reducing or eliminating the coffee-ring effect. Other groups have examined several parameters of metallic nanoparticle inks in view of their influence on the overprint geometry, including the solvent [33], particle size [35] and concentration [36], ink viscosity [37] and conductivity [38]. Apart from a proper ink and substrate, the printing parameters offer a wide range of adjustment of the line geometry. Since e-jet printing is based on electrohydrodynamically induced flows, most common process parameters are connected to the electric field applied for the ink deposition. These parameters include the voltage bias between the nozzle and the printing table, the amplitude and frequency of the voltage pulse, and on the distance between nozzle and substrate. Previous studies reported the printing of continuous Ag lines on silicon wafers [39] and glass substrates [40], with an overprint linewidth directly proportional to the amplitude of the voltage pulse. The influence of the pulse frequency was also examined in various regimes [41] and on various substrates, including highly insulating substrates [42]. Finally, a drastic change of linewidth and morphology was reported, while changing the standoff height, i.e., the nozzle–substrate distance [43]. Mantysalo et al. have conducted an extensive analysis of the effects of printing parameters on the Ag-nanoparticle ink overprints by developing a statistical model for droplet width as a function of four main parameters, such as print height, bias voltage, peak voltage and frequency [44]. Despite intensive research on the electrode deposition by the e-jet, the implementation of optimized overprints as conductive lines in electronic devices is still scarce. Development of new fabrication procedures and improvement of the electrical resistance without realization and analysis of the entire electronic device does not result in solving the most common and critical problems encountered [45,46]. In the case of OFETs, crucial aspects include contact resistance and the deposition of the source and drain electrodes [47]. In this work, we demonstrate fine geometry control of silver overprints printed by an ultraprecise e-jet. We investigate the influence of printing parameters on the linewidth and cross-section of sintered silver overprints and correlate the electrode morphology to their resistivity. The process parameters are optimized for single and multilayer overprints by performing detailed analysis of their geometrical features. To relate their properties with the device performance and finally to prove their potential in practical applications, the overprints are incorporated in OFETs as source and drain electrodes.

## 2. Materials and Methods 

### 2.1. Silver Nanopaste Ink

A silver nanoparticle (5–10 nm diameter) ink DGP 40LT-15C containing 31.04 wt.% Ag in triethylene glycol monoethyl ether (TGME) (Advanced Nano Products Co., Ltd., Sejong, Korea) was used for the inkjet printing of the conductive tracks and their further investigation as potential use in OFETs. The density, dynamic viscosity and surface tension of DGP 40LT-15C at 25 °C are 1.45 g/mL, 13.7cP and 34.65 mN/m, respectively. Conductivity of DGP 40LT-15C is 8.3–9.1 MS/m [48]. During all experiments the temperature of the DGP ink was kept at 25 °C.

### 2.2. Sample Preparation

Substrates consisted of heavily doped *n*-type Si wafers with a 300 nm thick SiO_2_ dielectric layer (capacitance of 11 nF cm^−2^) purchased from EL-CAT Inc. (Ridgefield Park, NJ, USA). Different preparation procedures of SiO_2_ surface modification were used (Table 1). All substrates were cleaned in a sequence of sonication baths in acetone and isopropyl alcohol (iPrOH). In addition, some samples were cleaned in chloroform (CHCl_3_), or treated with oxygen plasma (Plasma) for 5 min (Atto, Diener Electronic GmbH & Co KG, Ebhausen, Germany). Two other batches of SiO_2_ samples were, after sonication in isopropyl alcohol, chemically modified by either trichloro(octyl)silane (OTS) or hexamethyldisilazane (HMDS) monolayers. Prior to chemisorption of OTS or HMDS, the SiO_2_ substrates were cleaned in piranha solution. In the case of OTS functionalization, the substrates were immersed in μM solution of OTS in a 1:4 v/v mixture of chloroform and *n*-hexane. The substrates were kept in the OTS solution for 30 min and then rinsed with chloroform in an ultrasonic cleaner and dried with nitrogen. In the case of HMDS functionalization, the substrates were kept in HMDS vapor inside a closed chamber at a temperature 135 °C for 12 h, then rinsed with chloroform in an ultrasonic cleaner and finally dried with nitrogen.

Acetone, isopropyl alcohol and chloroform were purchased from Avantor Performance Materials Poland S. A. (Gliwice, Poland) and used as received. Trichloro(octyl)silane, *n*-hexane and hexamethyldisilazane were purchased from Sigma Aldrich and used as received.

### 2.3. Contact Angle Measurements

The static contact angle was determined using an OCA 15EC Goniometer from Data Physics Instruments GmbH, Filderstadt, Germany. Measurements were performed on SiO_2_ substrates with various surface treatments, as described in Section 2.2. For each sample the contact angle values were recorded at four different locations of the substrate and averaged. The contact angle of water was also measured for all types of substrates tested during this study as a reference. The droplet volume was 5 µL for the tests with both liquids and the accuracy of the measurements was ±0.5°.

### 2.4. Inkjet Printing

The printing was performed using the SIJ-S050 SuperInkjet printer (SIJ Technology, Inc., Tsukuba, Japan). SIJ-S050 is an electrohydrodynamic printer, which uses an electric field between the printer stage and the printing nozzle (3–4 μm inner aperture) to deposit small (~fL range) ink volumes. In order to eject the droplet from the capillary nozzle an electric pulse in the desired waveform was generated. Triangular waveform was used throughout the reported experiments. The amplitude and frequency of the pulse were modulated by the operator, and were chosen as basic control parameters in this study. During the printing process the nozzle remained static and the substrate was moved by a computer-controlled moving stage at constant speed of 1 mm/s. The printing height (the substrate-nozzle distance) was kept constant at around 50 μm. The optimal electric field to induce ejection of the DGP ink was set at *B* = 200 V, where *B* stands for the constant bias voltage between nozzle and sample stage. In addition, the amplitude (*A*) and peak of the alternating current (AC) voltage was set between 225 V and 300 V, to control the ejected ink volume. Applying *A* > 300 V in the studied frequency regime (i.e., between *f* = 100 Hz and *f* = 1000 Hz) resulted in ejection of a too high DGP ink volume preventing from fine control of the overprints shape. But applying too low *A* did not lead to ejection of any ink.

Geometrical and electrical characterization of the overprints was performed after the thermal annealing/sintering process. In order to sinter the deposited ink and to remove the TGME solvent and the capping agents, the overprints were heated at 150 °C for 1 h. In the case of multiple overprints (*n* > 1), the thermal treatment was performed after the last layer was printed.

### 2.5. Geometrical Characterization of the Overprints

**Figure 1:**
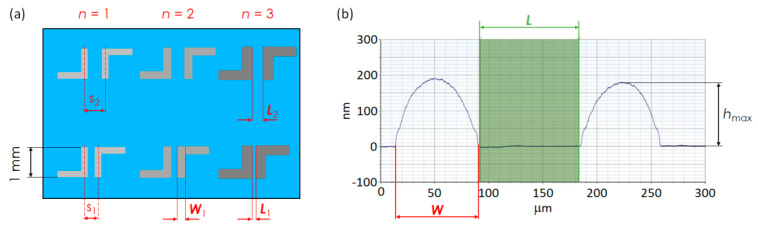
(**a**) Schematic top view representation of DGP overprints with various interline spacing (*s*) and different number of printing passes (*n*). (**b**) Exemplary cross-section profile underlining the geometrical parameters *W*, *L* and *h*_max_.

Width (*W*) of the overprints was estimated from the microscopy images recorded with the Omano OM349P optical microscope (Microscope LLC, Roanoak, VA, USA) and from the surface profilometry studies. Thickness of overprints was estimated with the DektakXT stylus profiler (Bruker Ltd., Coventry, UK). The maximum height (*h*_max_) represents the arithmetic average of maximum height of three different scans of the sample. Figure 1a depicts a typical sample in top view with both inputs (*s*, *n*) and outputs (*W*, *L*) geometrical parameters. Figure 1b shows an exemplary surface profile recorded by DektakXT, underlining *W*, *L* and *h*_max_. The cross-section area of the overprints was further used for calculations of the resistivity from the two-point probe measurements.

### 2.6. Resistivity Measurements of the Overprints

Resistivity (*ρ*) was estimated from the resistance measurements and geometrical parameters of the overprints (length and cross-section). Resistance was measured using a two-point probe method with a Keithley 2634B source meter (Keithley Instruments, Inc., Cleveland, OH, USA). The resistivity represents the average of 10 different overprints.

### 2.7. Organic Field-Effect Transistors

Donor-acceptor polymer poly[2,5-(2-octyldodecyl)-3,6-diketopyrrolopyrrole-alt-5,5-(2,5-di (thien-2-yl)thieno[3,2-b]thiophene)] (DPP-DTT) was purchased from Ossila Ltd., Sheffield, UK. DPP-DTT was chosen due to its high stability in ambient conditions. It was dissolved in chloroform (spectroscopic grade) and stirred at 50 °C for 1 h. The solution was filtered with a standard polyvinylidene fluoride (PVDF) syringe filter (40 μm). The Si/SiO_2_ substrates with printed silver electrodes were functionalized with HMDS prior to the deposition of semiconductor polymer film. DPP-DTT thin films were fabricated by means of spin coating at 3000 RPM for 30 s and angular acceleration of 3000 RPM/s. Afterwards, the samples were annealed at 200 °C for 15 min inside a glovebox (N_2_ atmosphere), to remove the remaining solvent. OFET measurements were performed by using a Keithley 2634B source meter. Output and transfer characteristics were measured in ambient atmosphere in the V_DS_ and V_G_ range from 10 V to −40 V. The charge carrier mobility for holes was derived from the transfer characteristics in the saturation regime using standard equations described elsewhere [49]. Five to 10 OFETs were tested for each sample.

## 3. Results

### 3.1. Role of Surface Energy

One of the crucial factors for printing is the wettability of the ink on the printed surface, because this influences the quality of the overprints and the overall process reliability. Since the printed structures are created by ink droplets, contact angle measurements are an important tool to evaluate the ink–substrate interactions. Fabrication of conductive tracks of sufficiently low electrical resistivity requires continuous overprints with homogenous shapes. To ensure reliability of the printed tracks, straight edges and uniform cross-section of the electrodes are required, especially for a well-defined constant channel length (*L*) between source and drain contacts of an OFET. Moreover, a smooth shape of the electrodes is important to minimize variations of the interfacial morphology at the metal/semiconductor interface in bottom contact OFETs. Both factors can result in a non-homogenous distribution of the electric field, which can reduce the device performance [50]. To control the quality of the overprints, the influence of surface energy on the DGP wettability was firstly studied. In all cases, sessile droplets deposited on various surface modifications revealed symmetric and regular shapes. The contact angles of H_2_O and DGP for different surface treatments are summarized in Table 1.

**Figure 2:**
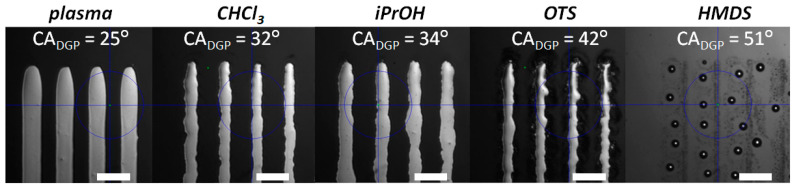
Optical microscope images of DGP overprints on SiO_2_ surface with different types of treatment. Scale bar equal 100 μm.

The contact angle for DGP shows a similar trend as the water reference. The values for the TGME-based DGP ink are smaller, indicating slightly improved wettability in comparison to water. Since TGME is a polar solvent, hydrophobic surfaces do not provide good wettability for the ink. OTS- and HMDS-modified surfaces exhibit the largest contact angle of 42° and 51°, respectively. The substrates washed with CHCl_3_ and iPrOH reveal smaller values of 32° and 34°, respectively. The smallest contact angle of 25° was found for the plasma-treated hydrophilic surface leading to the best wettability of the DGP ink. The effect of surface modification on the morphology of the printed DGP tracks is shown in Figure 2, where multiple lines of single DGP overprints are presented on differently modified surfaces. A very high contact angle of DGP on HMDS results in dewetting of the ink and lack of continuous overprints. In the case of OTS modification, the DGP ink forms continuous lines, however, with saw-like edges. We postulate a limit in contact angle between 40° and 50° for the wettability of the DGP ink. This is supported by previous studies which reported isolated ink drops and no continuous lines of DGP on polydimethylsiloxane (PDMS) substrates at a contact angle of 55.7°, regardless the drop spacing [51]. By contrast, if the contact angle of DGP is low enough, as in the case of polyethylenenaphthalate (PEN) film (~27°), wetting of the substrate allows uniform overprints to be obtained [52].

The overprints of DGP on surfaces treated with CHCl_3_ and iPrOH appear comparable to each other, in agreement with their close contact angles. Nevertheless, both cases also exhibit irregular shapes with edges moderately better defined than on OTS. Contrary to the other presented surfaces, the DGP overprints on plasma-treated substrates reveal linear shapes of the edges. Treatment of SiO_2_ surfaces with plasma does not only clean the surface of adsorbed contaminants [53], but also provides an activation of the surface. This activation can lead to the modification of surface morphology (i.e., roughness) [54], or can result in more uniform wetting properties, manifested by homogenous spreading of the ink, like in the case of DGP. From all studied samples, the plasma-treated SiO_2_ is the most suitable for inkjet printing of DGP electrodes and, therefore, was chosen for further studies in this work.

### 3.2. Influence of Printing Parameters on Geometry and Resistivity of the Overprints

In the previous section, the influence of the surface energy on the ink wettability was examined. In this section we investigate the effect of e-jet printing parameters related to the electric field on the printing quality to optimize the final electrode shape. A pair of L-shaped DGP lines was printed as source and drain electrodes in OFETs, with the active channel area between the vertical parts of printed lines.

#### 3.2.1. Amplitude of the Voltage Applied

**Figure 3:**
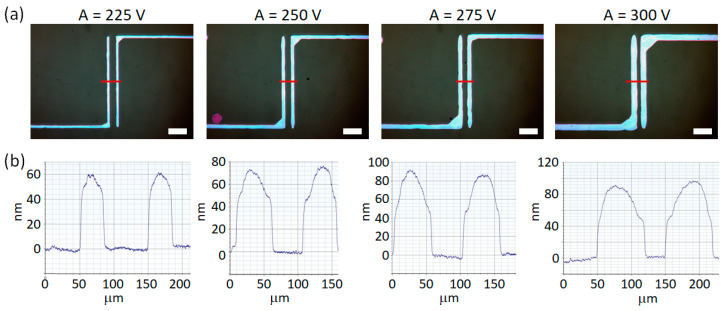
(**a**) Optical microscope images of DGP printed lines on plasma-treated SiO_2_ surface with different amplitudes (*A*). (**b**) Cross-section profiles of the respective overprints. *B* = 200 V, *f* = 500 Hz, *s* = 100 μm. Scale bar equal 200 μm.

The print resolution during inkjet printing is determined by the ink volume ejected from the nozzle. In e-jet printing, the droplet volume depends on the strength of the electric field (*E*) acting on the meniscus at the tip of the nozzle [19]. The strength of the electric field depends on the amplitude of the voltage applied (*A*) and on the distance between nozzle and substrate. Throughout the presented studies the nozzle–substrate distance was kept constant, while the influence of the voltage applied on the geometry of the overprints was investigated. Figure 3 shows optical microscopy images of four overprints with different values of *A*, equal to 225 V, 250 V, 275 V and 300 V, respectively. The base voltage (bias, *B*) was kept constant at *B* = 200 V. The overprints reveal continuous linear shapes with increasing width (*W*) for increased *A*, which is confirmed by surface profilometry measurements (cf. cross-section scans in Figure 3b).

Not surprisingly, the larger volume of deposited ink is reflected in an increased maximum height of the overprints, *h*_max_. More detailed analysis of the profiles reveals a rounded shape of the overprint upper part which evolves with the volume of the deposited ink. A small amount of the deposited ink results in a symmetric cross-section and similar shape of the overprints (*A* = 225 V, Figure 3b). With an increased *A* and larger volume of the deposited ink, the cross-section of both lines differ and the line shape becomes unsymmetrical (e.g., *A* = 275 V, Figure 3b). In particular, the point of maximum height is shifted from the center in the direction away from the prospective channel area. A possible reason of this distorted shape of the contact lines is an unsymmetrical wetting in two lateral directions related to the uneven charging of the substrate. The uneven charge can originate from the presence of another conductive line at the close proximity.

**Figure 4:**
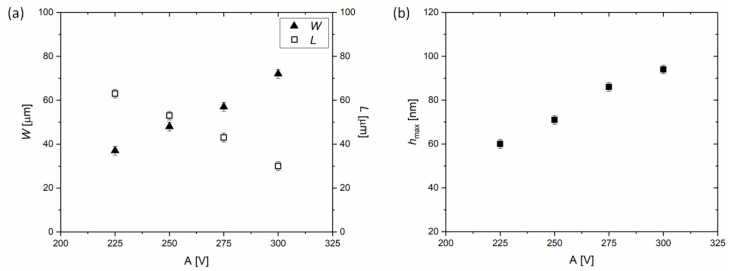
Influence of the amplitude of the applied voltage (*A*) on the geometrical features of the overprints on (**a**) width (*W*) and edge-to-edge distance, defining the channel length (*L*), and (**b**) maximum height of the overprints (*h*_max_). *B* = 200 V, *f* = 500 Hz, *s* = 100 μm.

A linear relation between the applied voltage and linewidth (*W*) as well as channel length (*L*) is found, as depicted by Figure 4a. The linewidth of the overprint *W* is controlled from 37 ± 2 μm for *A* = 225 V, to 71 ± 2 μm for *A* = 300 V. Figure 4b shows that the maximum height of the overprint (*h*_max_) increases also linearly with *A*. Thus, we conclude that it is possible to control not only the width, but in addition the thickness of DGP printed lines. By modulation of the amplitude voltage in the regime of *A* from 225 V to 300 V, a significant change of *h*_max_ from 60 ± 2 nm to 94 ± 2 nm, is achieved. Overall, the voltage amplitude is determined as a crucial parameter for the control of geometrical features of the overprints.

#### 3.2.2. Frequency of the Voltage Waveform

**Figure 5:**
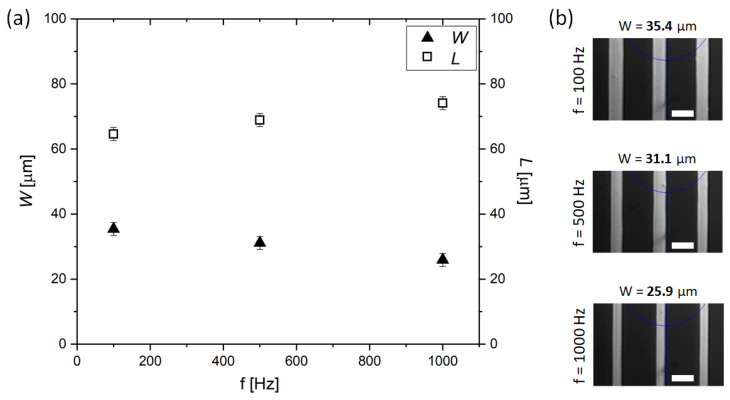
(**a**) Influence of the frequency of the voltage waveform (*f*) on the linewidth (*W*) of the overprints and on the resulting channel length (*L*), for s = 100 μm. (**b**) Optical microscope images for *f* = 100 Hz, *f* = 500 Hz and *f* = 1000 Hz, respectively. *A* = 300 V, *B* = 200 V, *s* = 100 μm. Scale bar equal 50 μm.

Another printing parameter which significantly influences the distribution of the printed ink is the frequency of the applied electric field (*f*). A triangular shape of the waveform was used throughout the presented studies. It is known from previous e-jet studies that the frequency of droplet ejection depends not only on the frequency of the voltage waveform, but also on the viscosity and electrical conductivity of the ink [38]. To study the size distribution of the DGP overprints by the change of the frequency of the applied electric field, the viscosity of DGP ink was kept constant since the printing was performed at ambient conditions (room temperature (RT) = 23 °C, humidity = 40%). Figure 5 shows the influence of the frequency of the triangular voltage change for *A* = 300 V on the width of the single layer overprint and on the resulting channel length. For *f* = 100 Hz, the width equals 35 ± 2 μm, and the channel length for the neighboring overprint at *s* = 100 μm, equals 65 ± 2 μm. For larger frequencies, i.e., *f* = 500 Hz or *f* = 1000 Hz, the width of the overprints decreases to 31 ± 2 μm or 26 ± 2 μm, respectively. The relative reduction of the overprint width is 11% and 26%, in the case of *f* = 500 Hz and *f* = 1000 Hz, representing significant narrowing. Simultaneously, frequency-driven decreasing of the overprint width results in the increase of the channel length to *L* = 69 ± 2 μm and *L* = 74 ± 2 μm, for intermediate (*f* = 500 Hz) and high (*f* = 1000 Hz) *f*, respectively. This confirms the possibility of controlling the geometrical features of the overprints by the frequency adjustment. However, it is clear that the influence of the frequency in the tested regime is minor compared to the magnitude of geometrical changes induced by the amplitude of the waveform. This observation is in agreement with previous report describing the influence of the four main e-jet print parameters (*A*, *B*, *f* and print height) on the droplet size. By using statistical analysis Mantysalo et al. have shown that the *A* has the strongest effect on the width of the droplets [44].

#### 3.2.3. Summary of the Geometrical Features of the Overprints

**Figure 6:**
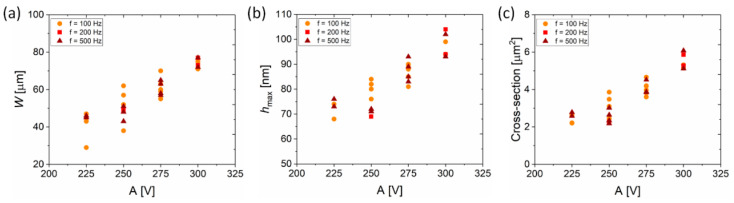
Summary of the geometrical features of the overprints fabricated with different *A* and *f*. (**a**) Width, (**b**) maximum height and (**c**) cross-section of the overprints increases for the higher voltage amplitude.

It is now well established that the printing parameters *A* and *f*, bear a significant influence on the geometry (*W* and *h*_max_) of the overprints. Since the cross-sectional features strongly depend on the printing parameters (Figure 3), a more general picture needs to be drawn. Width and maximum height are two geometrical parameters describing the maximum lateral and horizontal size of the overprints, but in order to determine correct resistivities of the printed DGP lines we need to establish a relation between the printing parameters and the cross-section of the overprints. Figure 6 summarizes the relation between the amplitude of the waveform and the width (Figure 6a), the maximum height (Figure 6b) and the cross-section (Figure 6c) of the respective overprints, for different *f*.

The data were collected from different samples, including the overprints fabricated with different frequencies and interline distances *s*, between 100 and 180 μm. There is a clear rising trend for each of the three geometrical features (*W*/*h*_max_/cross-section) as *A* is increased. The large sample size realized by testing multiple input variables (*A*, *f* and *s*) suggests that by choosing proper printing parameters a desired geometry of overprints can be printed.

#### 3.2.4. Resistivity of the Overprints

**Figure 7:**
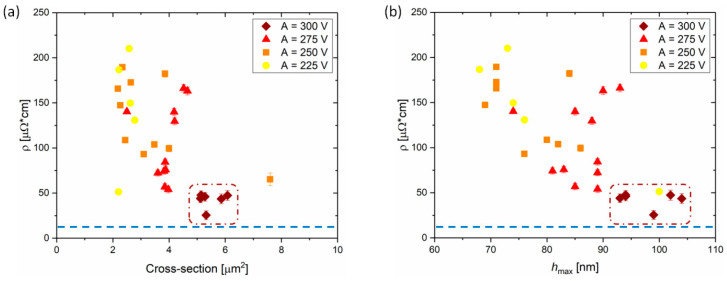
Summary of the resistivity measurements for DGP printed single layer overprints as a function of their cross-section (**a**) and their maximum height (**b**) for different *A*. The blue dashed lines represent the resistivity reported by the ink supplier.

Since printed silver tracks should serve as the electrodes in electronic devices, their resistivity was determined by two-point probe measurements. The resistivity was calculated from the resistance and the cross-section of the overprints. The length of the measured element was kept constant at 1 mm. The results of the resistivity measurements are summarized in Figure 7. Prior to the resistivity calculation the resistance was corrected by the contact resistance. Linear tracks of different lengths were fabricated and their resistance was extrapolated to the length equal to 0 (Figure S1, Supplementary Materials). The contact resistance from the two-point probe measurements was estimated to be 9.2 ± 0.4 Ω. This value was taken into account as an error value with respect to the total resistance, and it was treated as an error of the calculated resistivity of the single overprints (shown in Figure 7a,b as error bars).

All single layer overprints revealed a resistivity (*ρ*) in the range of 25–250 µΩ∙cm, i.e., larger than 12 µΩ∙cm reported by the ink manufacturer, which is highlighted by blue dashed lines in Figure 7. As noticed from Figure 7a, the overprints with smaller cross-section tend to show larger *ρ*, in comparison to the lines of larger cross-section. Overprints with cross-section smaller than 4 µm^2^, reveal a wide spread in resistivity, while for cross-sections above 4 µm^2^ the values remain almost constant at around 40–50 µΩ∙cm. From Figure 7a, we can conclude that a voltage amplitude above 275 V is needed for single layer overprints of low resistivity. The minimum resistivity of 25.5 ± 4.9 µΩ∙cm for the single layer overprints corresponds well with the previously reported resistivity of single DGP overprints on glass after similar thermal sintering [55].

In Figure 7b, the resistivity is presented as a function of maximum height of the overprint. For *h*_max_ above 90 nm, the resistivity is constant at around 50 µΩ∙cm, while below 90 nm the data-points are strongly spread. Analogically to the cross-section, *A* of at least 300 V is required to fabricate overprints with maximum height of at least 90 nm. This means that *h*_max_ is an alternative geometrical feature which enables us to monitor the resistivity and quality of the overprints.

By performing the analysis of several process parameters we established a relation between the geometry and resistivity of the printed single layer overprints. Two distinct regimes for the overprint resistance are evident and sufficiently large *A* is required to maximize the electrical characteristics which depend on the cross-section or maximum height of the overprints. In the next section we will demonstrate how to further optimize the electrical performance of the printed silver lines by applying multilayer printing.

### 3.3. Optimization of the Number of Overprints

Since we have demonstrated that the electrical resistance of the overprints increases significantly when their cross-section or *h*_max_ drops below the respective threshold, it appears particularly important to fabricate overprints with sufficiently large geometrical features. It was shown in previous sections that *A* is the most powerful parameter for modulating the overprint size. However, simply increasing the amplitude and thus the electric field needs to take into account the quality of the overprints. The linear shape of the overprint edges might be seriously disturbed by a momentum of the droplet impact with the substrate that is too high. Multilayer printing is an alternative way of deposition of a sufficient amount of conductive ink, and the fabrication of overprints with appropriate cross-section and height. To achieve precise geometry control of the overprints, we have performed systematic studies of multilayer printing of the DGP ink.

**Figure 8:**
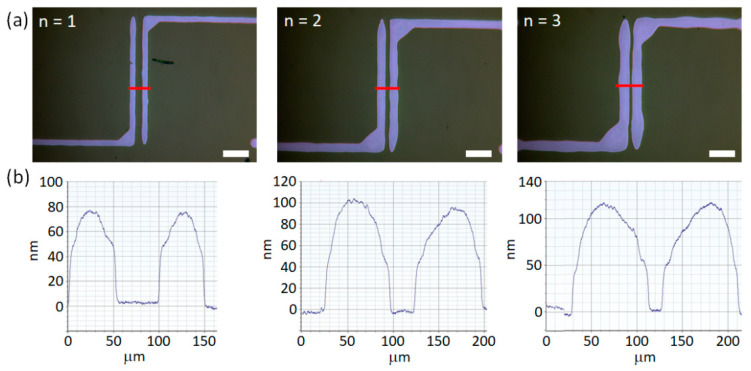
(**a**) Optical microscope images of DGP overprints on plasma-treated SiO_2_ surface with different number of printed layers (*n*). (**b**) Cross-section profiles of the respective overprints. *A* = 250 V, *B* = 200 V, *f* = 100 Hz, *s* = 100 µm. Scale bar equal 200 µm.

Figure 8a shows the optical microscopy images of single (*n* = 1), double (*n* = 2) and triple overprints (*n* = 3). The values of *A*, *B* and *f* were kept constant at 250 V, 200 V and 100 Hz, respectively. The overprints reveal continuous regular shapes with increasing width (*W*) for increased *n*, which is confirmed by cross-section scans shown in Figure 8b. The width of single, double and triple overprints was 50 ± 2 µm, 75 ± 2 µm and 82 ± 2 µm, respectively. Similar to applying larger amplitude, performing the additional printing cycle results in larger *h*_max_ of the overprint. The maximum height of single, double and triple overprints was 76 ± 2 nm, 100 ± 2 nm and 117 ± 2 nm, respectively. Comparing these values to previously established threshold of 90 nm we expect that the resistivity should drop when comparing overprint *n* = 1 with *n* = 2. This is further supported by the fact that the cross-section for single and double overprints is estimated from the profilometry scans to be equal 3.4 µm^2^ and 6.2 µm^2^, respectively.

**Figure 9:**
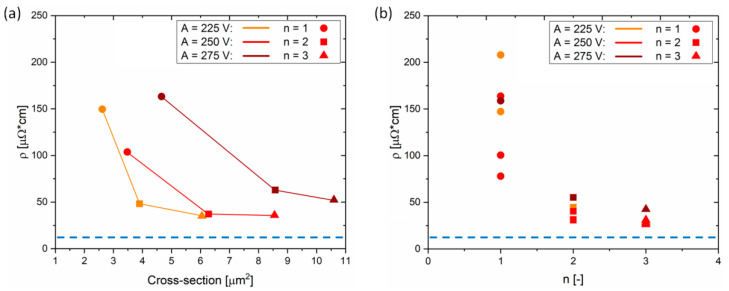
Summary of the resistivity measurements of single and multilayer overprints. (**a**) Two-point probe resistivity as a function of the overprint cross-section for different *A*. (**b**) Resistivity presented as a function of number of printed layers (*n*). The blue dashed lines represent the resistivity reported by the ink supplier. *B* = 200 V, *f* = 100 Hz.

Figure 9a shows the results of the resistivity measurements, for a series of single and multilayer overprints, as a function of overprint cross-section. Resistivities of the overprints discussed in Figure 8 are represented by red data-points. In agreement with the assumptions based on geometrical parameters *W* and *h*_max_, the resistivity of overprints drops for increased *n*, namely from 104 µΩ∙cm for *n* = 1, to 37 µΩ∙cm for *n* = 2 (circle and square data-points in Figure 9a). However, even though the third layer (*n* = 3) results in a noticeable increase of *W* and *h*_max_, the resistivity remains almost unchanged at 36 µΩ∙cm (triangle data-points in Figure 9a). The addition of the second layer of ink decreases the resistivity by a factor of 3, while printing the third layer does not improve the electrical properties significantly. This behavior may be also encountered when analyzing other series of overprints presented in Figure 9, for both smaller (*A* = 225 V) and larger (*A* = 275 V) amounts of ink deposited. Despite significant difference in resistivity of single and double layer DGP overprints, their surface profilometry studies (Figure S2, Supplementary Materials) did not reveal differences in their topography on a 1 mm length. The thickness of both overprints is similarly uniform along the overprint without abrupt variations. Therefore, fabrication of proper conductive paths of DGP on plasma-treated Si/SiO_2_ wafer requires deposition of a double layer and printing of subsequent layers only slightly improves the electrical properties of the overprints. Figure 9b summarizes resistivities of the printed silver lines versus the number of layers deposited. Contrary to the resistivity of single layer overprints which vary from tens to hundreds of µΩ∙cm (as shown before in Figure 7), printing of successive layers almost always ensures more constant values at *ρ* < 50 µΩ∙cm.

**Figure 10:**
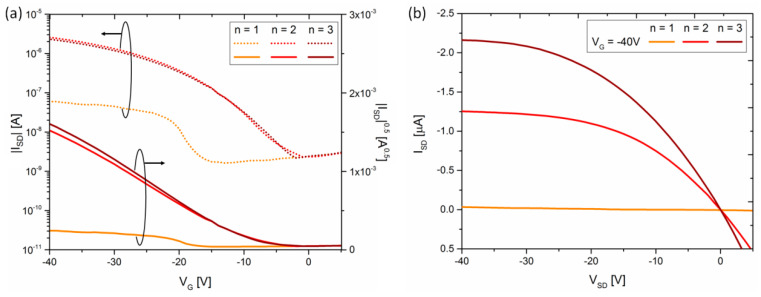
(**a**) Transfer and (**b**) output characteristics of bottom contact/bottom gate organic field-effect transistors (OFETs) with DPP-DTT (poly[2,5-(2-octyldodecyl)-3,6-diketopyrrolopyrrole -alt-5,5-(2,5-di(thien-2-yl)thieno[3,2-b]thiophene)]) as active layer, comprising single (*n* = 1), double (*n* = 2) and triple (*n* = 3) overprints as electrodes. V_SD_ = −40 V for transfer characteristics.

The ultimate step consisted of the application of the DGP overprints as source and drain electrodes in bottom contact/bottom gate OFETs. The resistivity change between single and multilayer electrodes was reflected also in the electrical performance of devices containing a similar active layer. A stable solution-processed conjugated polymer with high hole mobility (DPP-DTT, [56]) was used as the model system in these studies. Figure 10 shows typical transfer and output characteristics of OFETs based on spin-coated DPP-DTT comprising electrodes with a different number of printed DGP layers.

Transfer characteristics of OFETs exhibit a typical unipolar *p*-channel behavior (Figure 10a). However, the *I-V* curve of devices comprising single layer electrodes differ strongly from the ones with double or triple layer electrodes. The I_SD_ at V_SD_ = −40 V is almost two orders of magnitude higher for multilayer electrodes than for single layer ones. The average hole mobility extracted from OFETs with single layer electrodes is *µ_n1_* = 4.4 × 10^−3^ cm^2^ V^−1^ s^−1^, and increases by an order of magnitude for devices with multilayer electrodes to *µ_n2_* = 3.3 × 10^−2^ cm^2^ V^−1^ s^−1^ and *µ_n3_* = 4.0 × 10^−2^ cm^2^ V^−1^ s^−1^. The charge carrier mobilities for bottom contact/bottom gate OFETs comprising multilayer electrodes are slightly better than devices with thermally evaporated Ag electrodes for which *µ_Ag_* = 1.0 × 10^−2^ cm^2^ V^−1^ s^−1^ (Figure S3, Supplementary Materials). However, either printed or evaporated silver-based electrodes reveal mobilities smaller by a factor of 2–3 in comparison with bottom contact/bottom gate OFETs comprising Au electrodes reported previously [57]. The reason is most probably related to improperly aligned HOMO of DPP-DTT (−5.2 eV, [56]) and workfunction of printed DGP lines (−4.24 eV, [52]). In agreement with the significantly lower I_SD_ for *n* = 1, the on/off current ratio (*I_ON_/I_OFF_*) for OFETs comprising single layer electrodes reveals smaller values than for devices with multilayer electrodes. While the former exhibits *I_ON_/I_OFF_* inferior to 100, for the latter it exceeds 10^3^. In addition, the threshold voltage *V_th_* shifts from −13 V for OFET with *n* = 1 to around −5 V (−4 V), for *n* = 2 (*n* = 3).

Overall, we find that the charge carrier transport in thin films of DPP-DTT is limited by the injection of charge carriers in OFETs comprising single layer electrodes. This is further manifested in Figure 10b, which shows the output characteristics of OFETs with electrodes composed of a different number of DGP layers. The devices with double and triple DGP layers (*n* = 2 and *n* = 3, respectively) exhibit a clear linear region starting at V_SD_ = 0 V, and a successive saturation region at higher negative voltage. By contrast, the source-drain current for OFETs with single layer electrodes (Figure S4, Supplementary Materials) does not cross the abscissa at the origin and a superlinear behavior at low source-drain voltage is observed. This behavior is attributed to contact resistance between the source/drain electrodes and the semiconducting layer [58]. In addition, the output characteristics for single layer electrodes do not exhibit a clear saturation region in the investigated voltage range, and source-drain currents are two orders of magnitude smaller than for multilayer electrode devices. Altogether, we notice a qualitative and quantitative difference in the *I-V* characteristics of OFETs comprising single layer and multilayer electrodes, represented by a large difference in all key performance parameters, *µ*, *V_th_* and *I_ON_/I_OFF_*. Addition of the second layer leads to a great improvement in the electrical performance of the OFETs. The ink wetting on the plasma-treated substrate leads to the low thickness and finally to high resistivity of single-layer overprints. The percolation path for silver overprints with thickness smaller than the estimated *h*_max_ threshold of 90 nm hinders an efficient injection of charge carriers from the electrode to the active layer.

## 4. Conclusions

In this work we have optimized the printing parameters for the realization of conductive tracks, either by applying sufficiently large voltage amplitude or by deposition of multilayers. The influence of several printing parameters on the geometrical features of the silver overprints was investigated, confirming that the amount of deposited ink is crucial for the reliability of electrodes. The precise deposition of the DGP ink was achieved by combining the extraordinary jetting capabilities of the electrohydrodynamic printer with the adjusted wettability of the substrate. A detailed exploration of the relation between geometry and resistivity of printed tracks enabled us to establish threshold values for the cross-section and maximum height, above which resistivity dropped significantly.

Finally, it was shown that the geometry-dependent properties of the printed electrodes as source/drain bottom contacts in OFETs determine the device performance. A large contact resistance was observed for single layer DGP OFETs. Key performance parameters for OFETs (*μ*, *V_th_* and *I_ON_/I_OFF_*) differed by orders of magnitude for devices comprising single layer and multilayer electrodes. Control over the overprint geometry ensures an efficient injection of charge carriers from contacts to the active semiconducting film. The results presented here further deepen knowledge about the precise fabrication of electrodes, combining surface science and device engineering. In the near future, we plan to further optimize the fabrication of printed OFETs by including interlayers and performing patterning.

## Figures and Tables

**Table 1:** Summary of H_2_O and DGP contact angles (CA) for different surface treatments Si/SiO_2_ substrates.

	Surface Treatment	CA(H_2_O)	CA(DGP)
1	Plasma	32°	25°
2	CHCl_3_	63°	32°
3	iPrOH	62°	34°
4	OTS	82°	42°
5	HMDS	96°	51°

## Supplementary Materials

The following are available online at https://www.mdpi.com/1996-1944/13/21/4974/s1, Figure S1: The length-dependent resistance measurements for estimation of the contact resistance; Figure S2: Surface profile of single (*n* = 1, top) and double (*n* = 2, bottom) DGP overprints; Figure S3: (a) Transfer and (b) output characteristics of bottom contact/bottom gate OFETs with DPP-DTT as active layer, comprising thermally evaporated Ag electrodes. V_SD_ = −40 V for transfer characteristics; Figure S4: Output characteristics of bottom contact/bottom gate OFETs with DPP-DTT as active layer, comprising single (*n* = 1), double (*n* = 2) and triple (*n* = 3) overprints as electrodes.
