# Supplementary material for: Geometry Control of Source/Drain Electrodes in Organic Field-Effect Transistors by Electrohydrodynamic Inkjet Printing
Source: Materials (Basel). 2020 Nov 5;13(21):4974. doi: 10.3390/ma13214974 (PMC7663849; doi:10.3390/ma13214974)
Supplement: Supplementary file 1 [file materials-13-04974-s001.pdf]

materials

Supplementary Materials

Geometry Control of Source/Drain Electrodes in Organic Field-Effect Transistors by Electrohydrodynamic Inkjet Printing

Piotr Sleczkowski [1,*], Michal Borkowski [1], Hanna Zajaczkowska [1], Jacek Ulanski [1], Wojciech Pisula [1,2] and Tomasz Marszalek [1,2,*]

[1] Department of Molecular Physics, Faculty of Chemistry, Lodz University of Technology, Zeromskiego 116, 90-924 Lodz, Poland; michal.borkowski@dokt.p.lodz.pl (M.B.), hanna.zajaczkowska@dokt.p.lodz.pl (H.Z.), jacek.ulanski@p.lodz.pl (J.U.)
[2] Max Planck Institute for Polymer Research, Ackermannweg 10, 55128 Mainz, Germany; pisula@mpip-mainz.mpg.de
* Correspondence: piotrb.sleczkowski@gmail.com (P.S.); marszalek@mpip-mainz.mpg.de (T.M.)

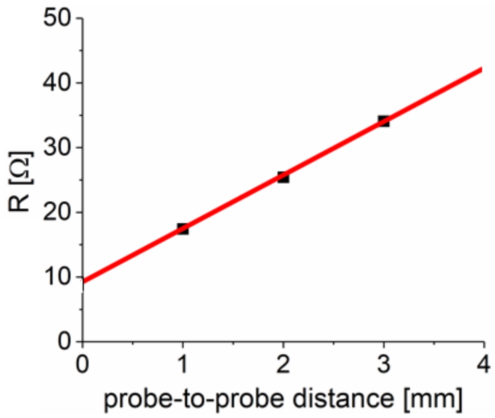

Figure S1. The length-dependent resistance measurements for estimation of the contact resistance.

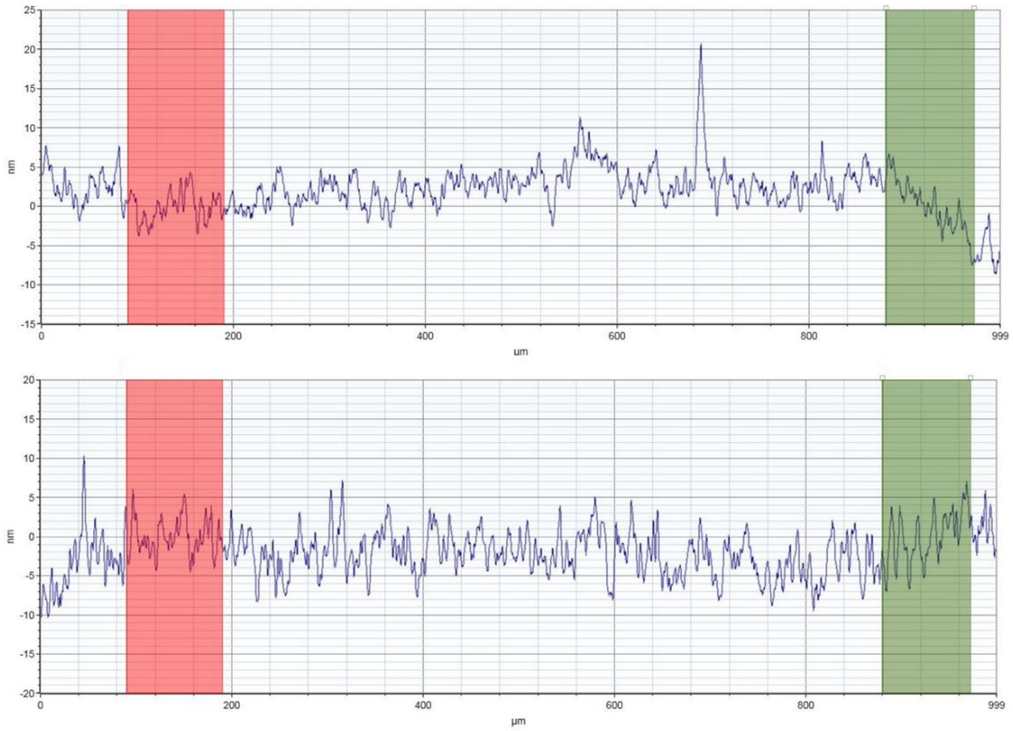

Figure S2. Surface profile of single (*n* = 1, **top**) and double (*n* = 2, **bottom**) DGP overprints.

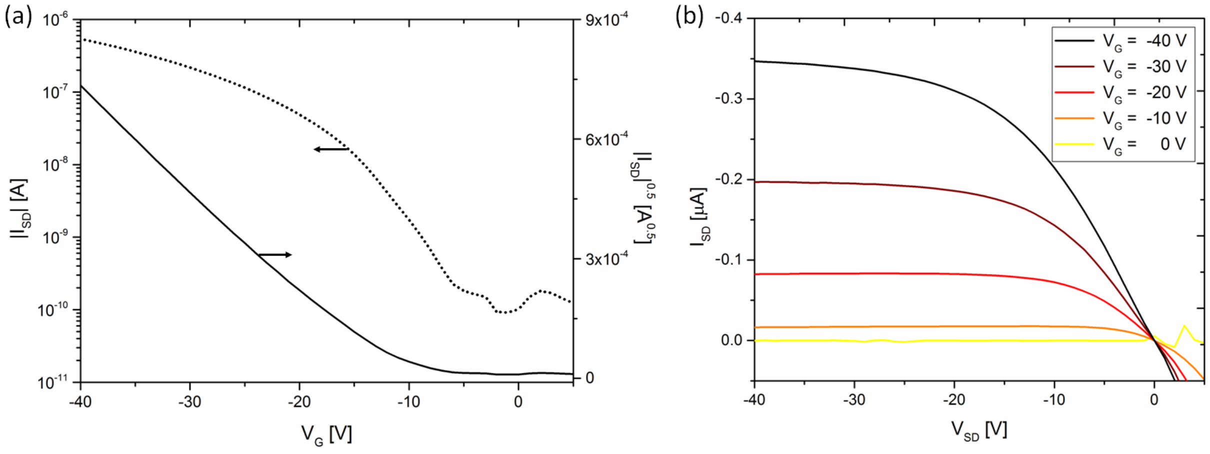

Figure S3. (**a**) Transfer and (**b**) output characteristics of bottom contact/bottom gate OFETs with DPP-DTT as active layer, comprising thermally evaporated Ag electrodes. V_{SD} = −40 V for transfer characteristics.

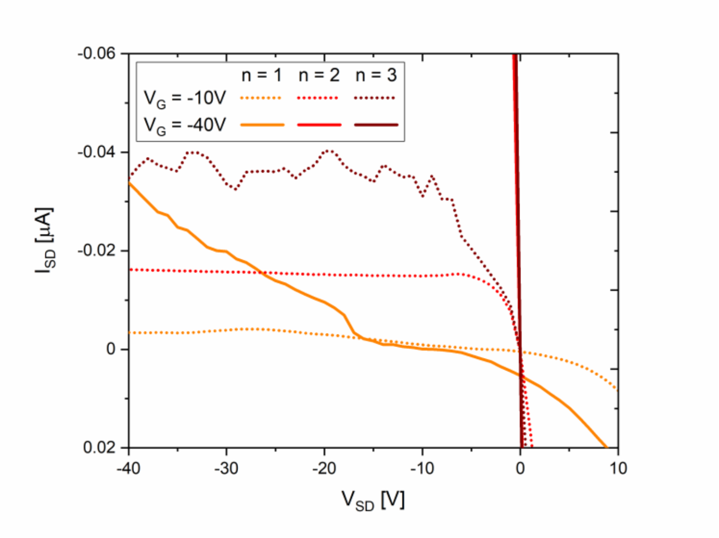

Figure S4. Output characteristics of bottom contact/bottom gate OFETs with DPP-DTT as active layer, comprising single (*n* = 1), double (*n* = 2) and triple (*n* = 3) overprints as electrodes.

© 2020 by the authors. Submitted for possible open access publication under the terms and conditions of the Creative Commons Attribution (CC BY) license (http://creativecommons.org/licenses/by/4.0/).
